# Folic acid-targeted iron oxide nanoparticles as contrast agents for magnetic resonance imaging of human ovarian cancer

**DOI:** 10.1186/s13048-016-0230-2

**Published:** 2016-03-29

**Authors:** He Zhang, Jingchao Li, Yong Hu, Mingwu Shen, Xiangyang Shi, Guofu Zhang

**Affiliations:** Department of Radiology, Obstetrics and Gynecology Hospital, Fudan University, No.419 Fangxie Road, Shanghai, 200011 P. R. China; College of Chemistry, Chemical Engineering and Biotechnology, Donghua University, Shanghai, 201620 P. R. China

**Keywords:** Ovarian cancer, Iron oxide nanoparticles, Folic acid, Targeting, Magnetic resonance imaging

## Abstract

**Background:**

Improved methods for the early and specific detection of ovarian cancer are needed.

**Methods:**

In this experimental study, we used folic acid (FA)-targeted iron oxide (Fe3O4) nanoparticles (NPs) as a T2-negative contrast agent for magnetic resonance (MR) imaging to accurately detect ovarian cancer tissues in an intraperitoneal xenograft tumor model. Human serous ovarian cell line (Skov-3), with overexpressed FA receptors, was chosen as the targeted tumor cell mode. For in vivo experiments, the cells were injected intraperitoneally into nude mice to produce intraabdominal ovarian cancers. FA-targeted and non-targeted Fe_3_O_4_ NPs were prepared.

**Results:**

FA-targeted Fe_3_O_4_ NPs with a mean size of 9.2 ± 1.7 nm have a negligible cytotoxicity to human serous ovarian cell line (Skov-3). Importantly, the results of cellular uptake suggested that FA-targeted Fe_3_O_4_ NPs have a targeting specificity to Skov-3 cells overexpressing FA receptors. FA-targeted Fe_3_O_4_ NPs could be specifically localized by magnetic resonance (MR) imaging to the intraperitoneal human ovarian carcinoma tissues, as documented by a statistically significant difference (*p* = 0.002, *n* = 3) in T_2_ signal intensities of xenograft tumor tissues when injected with FA-targeted and non-targeted Fe_3_O_4_ NPs at 4 h post-injection.

**Conclusion:**

FA-targeted Fe_3_O_4_ NPs appear to be promising agents for the detection of human ovarian carcinoma by MR imaging, and possibly also for the hyperthermal treatment of the tumors.

## Background

Ovarian cancer is the sixth most commonly diagnosed cancer in the world, accounting 4 % of all cancers in women [1], and it is the leading cause of death from gynecologic malignancies in the western world [2, 3]. Most ovarian cancers are first diagnosed in an advanced stage because patients’ symptoms may be minimal or nonspecific and no reliable biomarkers are available [4]. Tumor-debulking surgery is the first choice of management for most patients with ovarian cancer [5], but most ovarian cancers recur after surgery and are intractably drug resistant [6]. Therefore, although some advances in cytoreductive surgery and case-effective chemotherapy have been made in the last decade, the prognosis for ovarian cancer, especially for epithelial ovarian cancer still is limited.

In most tertiary medical centers, magnetic resonance (MR) imaging is generally performed for imaging assessment of complex ovarian masses [7, 8] that are indeterminate on either palpation or ultrasonography because of MR’s superb soft-tissue resolution and lack of radiation. The MR diagnostic criteria for ovarian malignancies are based on morphology: thick septum, vegetations, ascites, lymphadenopathy, and vividly enhancing solid component, which are features well described in numerous reports [8, 9]. However, identification of the tumor tissues at an early stage with available imaging modalities still possesses a great challenge for both radiologists and clinicians.

Recent advances in nanoscience and nanotechnology have enabled the development of various contrast agents for MR imaging applications, such as Gd (III)- or Mn (II)-based T_1_ MR contrast agents [10, 11] and magnetic iron oxide nanoparticle (Fe_3_O_4_ NPs)-based T_2_ MR contrast agents [12–14]. The Fe_3_O_4_ NPs are the most commonly used magnetic materials for various biomedical applications [15–18]. But, few reports on the application of Fe_3_O_4_ NPs for the diagnosis of ovarian cancer have been published.

Folic acid (FA) receptors as single-chain glycoproteins with high specific affinity for FA are highly overexpressed on various malignant tumors, including human ovarian cancer [19]. The over-expression of FA receptors on malignant tumor tissues can be exploited as a specific targeting ligand since most healthy tissues have little FA receptors expression [20]. This targeting strategy has the potential for diagnostic and therapeutic application in a wide variety of cancers [21, 22].

In this research, we used FA-targeted Fe_3_O_4_ NPs as T_2_-negative contrast agents for in vivo MR imaging of ovarian cancer in an intraperitoneal xenograft tumor model. To the best of our knowledge, this is the first reported application of FA-targeted Fe_3_O_4_ NPs in MR imaging diagnosis of ovarian cancer.

## Methods

### Synthesis and characterization techniques

FA-targeted Fe_3_O_4_ NPs were synthesized and characterized according to our previous work [23]. Non-targeted Fe_3_O_4_ NPs were synthesized by the same methods, except for the use of *m*PEG-COOH in the PEGylation step instead of FA-PEG-COOH.

Branched polyethyleneimine (PEI, Mw = 25,000)-coated Fe_3_O_4_ NPs (Fe_3_O_4_@PEI NPs) were synthesized via a reduction route. FeCl_3_ · 6H_2_O (1.3 g) was dissolved in 20 mL water, and placed into a 250 mL three-necked flask. Under vigorous stirring, the solution was bubbled with nitrogen atmosphere for 15 min, then 10 mL freshly prepared sodium sulfite solution (0.2 g) was added slowly into the flask. 30 min later, 5 mL PEI (0.5 g) and 2 mL ammonia (25 %) was added into the flask successively. The reaction mixture was vigorously stirred for 30 min at 60 ~ 70 ^o^C, and then at room temperature for another 1.5 h. The product (Fe_3_O_4_@PEI NPs) was magnetically collected and washed 3 times with water. Finally, the sample was centrifuged (8000 rpm, 10 min) to remove the aggregation and larger particles.

An aqueous solution of Fe_3_O_4_@PEI NPs (110 mg, 35 mL) was precipitated by virtue of an external magnet and re-dispersed in 20 mL DMSO. Another solution of 38.5 mg activated FA-PEG-COOH or *m*PEG-COOH in 2 mL DMSO was added dropwise into the above DMSO solution of Fe_3_O_4_@PEI NPs and kept shaking for 3 d. The formed products were collected by magnetic separation and washed with DMSO for 3 times to remove excess reactants. Finally, the amino groups on the surface of the particles were acetylated by reaction with acetic anhydride. Briefly, triethylamine (493 μL) was added into the aqueous solution of raw product of Fe_3_O_4_@PEI-PEG-FA NPs or Fe_3_O_4_@PEI-*m*PEG NPs under vigorous shaking using a shaker at room temperature. After 30 min, acetic anhydride (402 μL) was dropwise added into the above mixture solution and the reaction was continued for 1 d. After several times magnetic separation/washing/dispersion steps to remove excess reactants and by-products, the final products (FA-targeted Fe_3_O_4_ NPs and non-targeted Fe_3_O_4_ NPs) were obtained, re-dispersed in water and stored under 4 ^o^C for further use.

A JEOL 2010 F transmission electron microscopy (TEM, JEOL, Tokyo, Japan) was used to characterize the morphology of the FA-targeted Fe_3_O_4_ NPs and non-targeted Fe_3_O_4_ NPs at an operating voltage of 200 kV. A dilute particle suspension of the sample in water (10 μL) was deposited onto a carbon-coated copper grid and dried in air before measurements. The effect of MR imaging for FA-targeted and non-targeted Fe_3_O_4_ NPs was evaluated with a 1.5 Tesla MR imaging machine (Siemens Avanto, Erlangen, Germany). Samples were diluted with water to have different Fe concentrations in the range of 0.005–0.08 mM before measurements. The T_2_-weighted imaging parameters with turbo spin echo sequence were set as follows: point resolution = 156 mm × 156 mm, section thickness = 1.5 mm, TR = 4000 ms, TE = 85 ms, bandwidth (Hz) = 260, number of excitation = 1, and voxel size = 1.1 × 1.1 × 4.0 mm.

### Cell culture

Skov-3 cells was obtained from the Shanghai Key Laboratory of Female Reproductive Endocrine Related Diseases (Shanghai, China). Skov-3 cells were grown in FA-free RPMI-1640 medium supplemented with 10 % fetal bovine serum (FBS), penicillin (100 U/mL) and streptomycin (100 μg/mL) at 37 °C and 5 % CO_2_.

### Cytotoxicity of FA-targeted Fe_3_O_4_ NPs and non-targeted Fe_3_O_4_ NPs

The 3-(4,5-dimethylthiazol-2-yl)-2,5-diphenyltetrazolium bromide (MTT) viability assay was carried out to evaluate the cytotoxicity of the FA-targeted Fe_3_O_4_ NPs and non-targeted Fe_3_O_4_ NPs. Briefly, 1 × 10^4^ Skov-3 cells were seeded into each well of 96-well cell culture plates with 200 μL regular RPMI-1640 medium and cultured at 37 °C and 5 % CO_2_ overnight to bring the cells to confluence. Next, the medium in each well was discarded carefully and 200 μL of fresh medium containing phosphate-buffered saline (PBS), FA-targeted Fe_3_O_4_ NPs or non-targeted Fe_3_O_4_ NPs at the Fe concentration of 0.5 to 1.0 mM was added. After 24 h incubation at 37 °C and 5 % CO_2_, 20 μL MTT solution (5 mg/mL in PBS buffer) were added to each well to reveal the viable cells. After further incubation for 4 h at 37 °C and 5 % CO_2_, the medium was carefully removed, and DMSO (200 μL) was added to dissolve the formazan grains. The absorbance value of each well was measured with a microplate reader at 450 nm wavelength.

### Cellular uptake of FA-targeted Fe_3_O_4_ NPs and non-targeted Fe_3_O_4_ NPs

To qualitatively confirm the cellular uptake of Fe_3_O_4_ NPs by Skov-3 cells, the cells was stained with Prussian blue. In brief, 5 × 10^5^ cells were seeded into each well of 24-well cell culture plates. After overnight incubation at 37 °C and 5 % CO_2_ to bring the cells to 80 % confluence, the medium was replaced with fresh medium containing PBS buffer (control), FA-targeted Fe_3_O_4_ NPs, or non-targeted Fe_3_O_4_ NPs at the Fe concentrations of 0.2 and 0.4 mM. The cells were continuously incubated for another 4 h. The cells were then washed three times with PBS, fixed with p-formaldehyde solution at 4 °C for 15 min, and stained with Prussian blue reagent (potassium ferrocyanide [1 g] dissolved in water [9 mL] mixed with 36–38 % HCl [1 mL]) at 37 °C for 30 min. The cells were imaged with a Leica DMIL LED inverted-phase contrast microscope.

The Leeman Prodigy inductively coupled plasma-optical emission spectroscopy (ICP-OES, Hudson, NH, USA) also was used to quantify the cellular uptake of the Fe_3_O_4_ NPs by Skov-3 cells. The Skov-3 cells were seeded into 12-well plates with a density of 1 × 10^6^ cells/well. After overnight incubation to bring the cells to confluence, the medium was discarded carefully, and 1 mL fresh medium containing PBS buffer (control), FA-targeted Fe_3_O_4_ NPs or non-targeted Fe_3_O_4_ NPs at Fe concentrations of 0.2 and 0.4 mM was added. The cells were further incubated at 37 °C and 5 % CO_2_ for 4 h. The medium was then removed. The cells were washed with PBS buffer four times, trypsinsized, collected, and suspended in 1 mL PBS buffer. The cell numbers in each sample were estimated with a hemocytometer. For the cellular uptake assay, the cells were centrifuged (1000 rpm, 5 min), collected, and lysed with an aqua regia solution (0.5 mL) for 12 h. The Fe content was determined by ICP-OES after the samples were diluted 2 times with PBS.

### In vivo targeted MR imaging of tumors

Four-week-old female BALB/c nude mice (Shanghai Cancer Institute, Shanghai, China) were treated according to protocols approved by the Ethical Committee of Obstetrics and Gynecology Hospital ([2007]-No. 6), Fudan University. The nude mice (three mice in each group) were injected intraperitoneally with 1 × 10^6^ Skov-3 cells/mouse at a site 1 cm left of the midline. Two weeks later, the mice were anesthetized with an intraperitoneal injection of pentobarbital sodium (40 mg/kg). After that, 200 μL of FA-targeted Fe_3_O_4_ NPs or non-targeted Fe_3_O_4_ NPs (0.6 mg Fe) were delivered into the mice via the tail vein. MR scans were performed before injection and 0.5, 1, 2, and 4 h after injection of the particles. A 1.5 T clinical MR system was used with a custom-built rodent receiver coil (Chenguang Med Tech, Shanghai, China). The sequence parameters were set as following: Axial fat-suppressed T2WI (FS T2WI), point resolution = 156 mm × 156 mm, TR/TE: 8000/83 ms, thickness: 2 mm, field of view: 50 mm, voxel size: 1.4 × 1.4 × 1.9 mm, flip angles: 150 °. Signal intensity in the tumors at each time point was measured and recorded.

### Statistical analysis

Quantitative data were expressed as mean ± standard deviation (SD). Means were compared by use of unpaired two-sided Student’s *t*-test. The data are indicated with (*) for *p* < 0.05, (**) for *p* < 0.01 and (***) for *p* < 0.001.

## Results

### Synthesis and characterization techniques

**Fig. 1:**
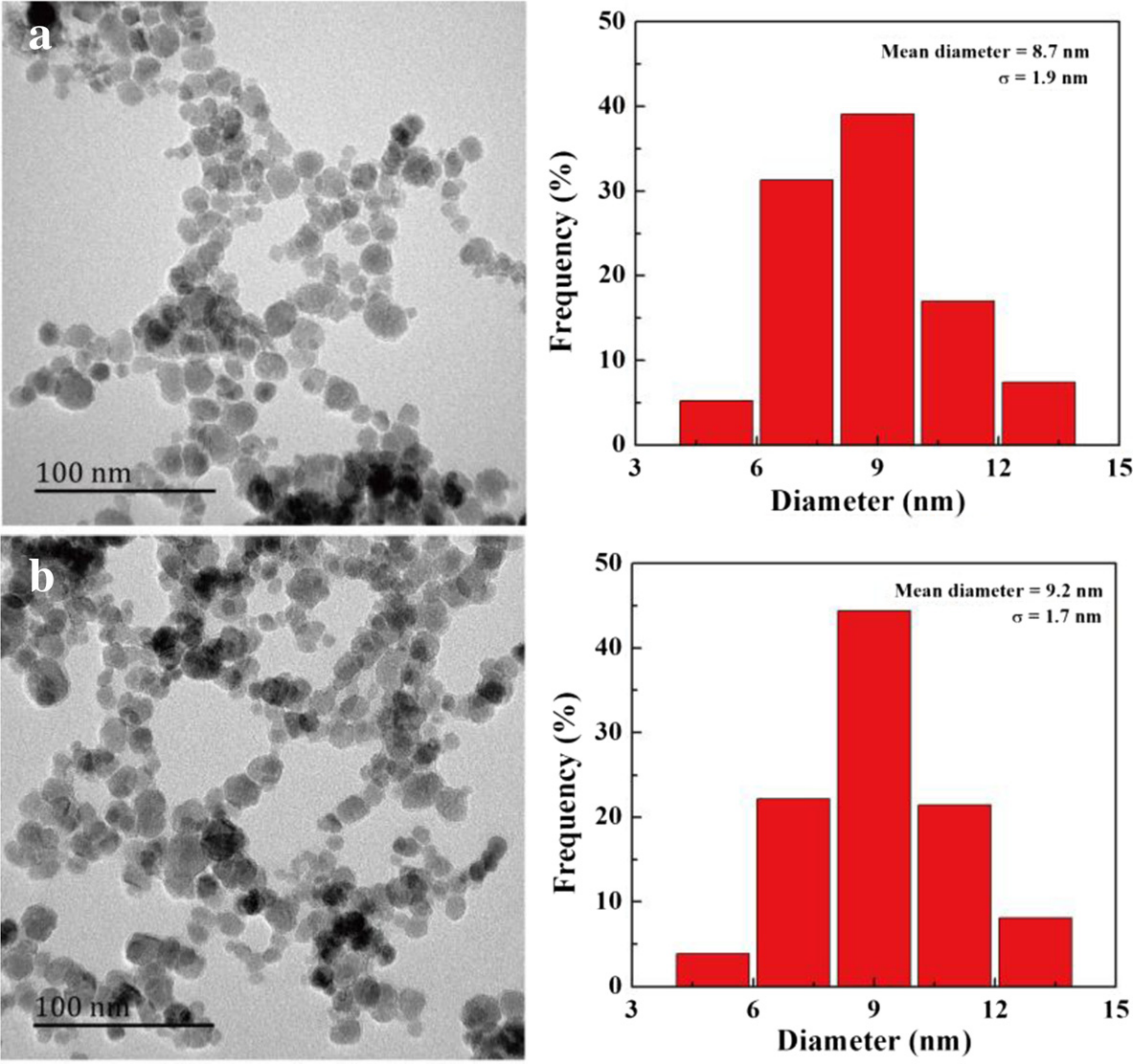
TEM micrographs and size distribution histograms of non-targeted Fe_3_O_4_ NPs (**a**) and FA-targeted Fe_3_O_4_ NPs (**b**)

**Fig. 2:**
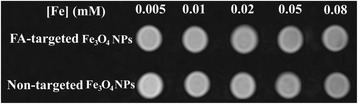
The T_2_-weighted MR images of the FA-targeted Fe_3_O_4_ NPs and non-targeted Fe_3_O_4_ NPs at different Fe concentrations

**Fig. 3:**
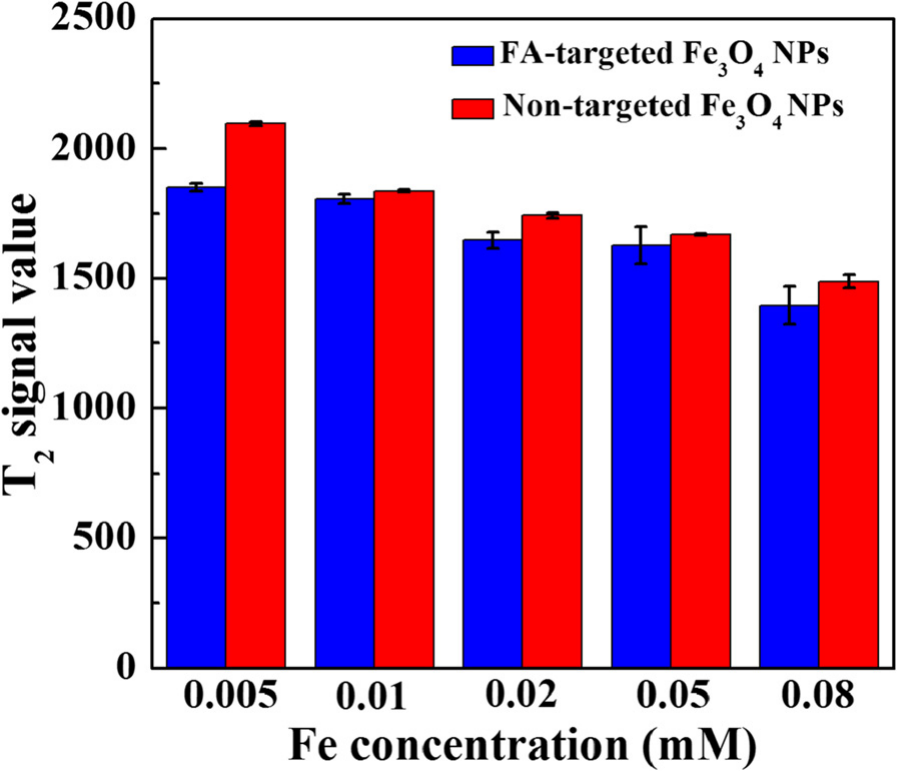
The T_2_ signal values of the FA-targeted Fe_3_O_4_ NPs and non-targeted Fe_3_O_4_ NPs at different Fe concentrations

The morphology of the FA-targeted Fe_3_O_4_ NPs and non-targeted Fe_3_O_4_ NPs was characterized by TEM (Fig. 1). It can be seen that the NPs with a spherical or quasi-spherical shape have a quite uniform size distribution and a polymer shell on their outer surface. The mean size was measured to be 8.7 ± 1.9 nm for non-targeted Fe_3_O_4_ NPs (Fig. 1a) and 9.2 ± 1.7 nm for FA-targeted Fe_3_O_4_ NPs (Fig. 1b), respectively. The T_2_-weighted MR effect of the NPs was evaluated by use of a 1.5 T MR system. Fe_3_O_4_ NPs decreased the MR signal intensity in relation to increasing Fe concentration for both FA-targeted Fe_3_O_4_ NPs and non-targeted Fe_3_O_4_ NPs (Fig. 2). The T_2_ signal intensities of FA-targeted Fe_3_O_4_ NPs at the given Fe concentrations were 1851 ± 14, 1808 ± 18, 1648 ± 30, 1628 ± 71, and 1395 ± 73. The T_2_ signal values in non-targeted Fe_3_O_4_ NPs at each given Fe concentration were 2094 ± 28, 1838 ± 14, 1742 ± 10, 1667 ± 2 and 1487 ± 26, respectively (Fig. 3). Based on the measured T_2_ relaxation time, the r_2_ relaxivity of FA-targeted Fe_3_O_4_ NPs and non-targeted Fe_3_O_4_ NPs was calculated to be 475.92 and 545.70 mM^−1^s^−1^, respectively according to our previous works [23].

### Cytotoxicity assay of FA-targeted Fe_3_O_4_ NPs and non-targeted Fe_3_O_4_ NPs

**Fig. 4:**
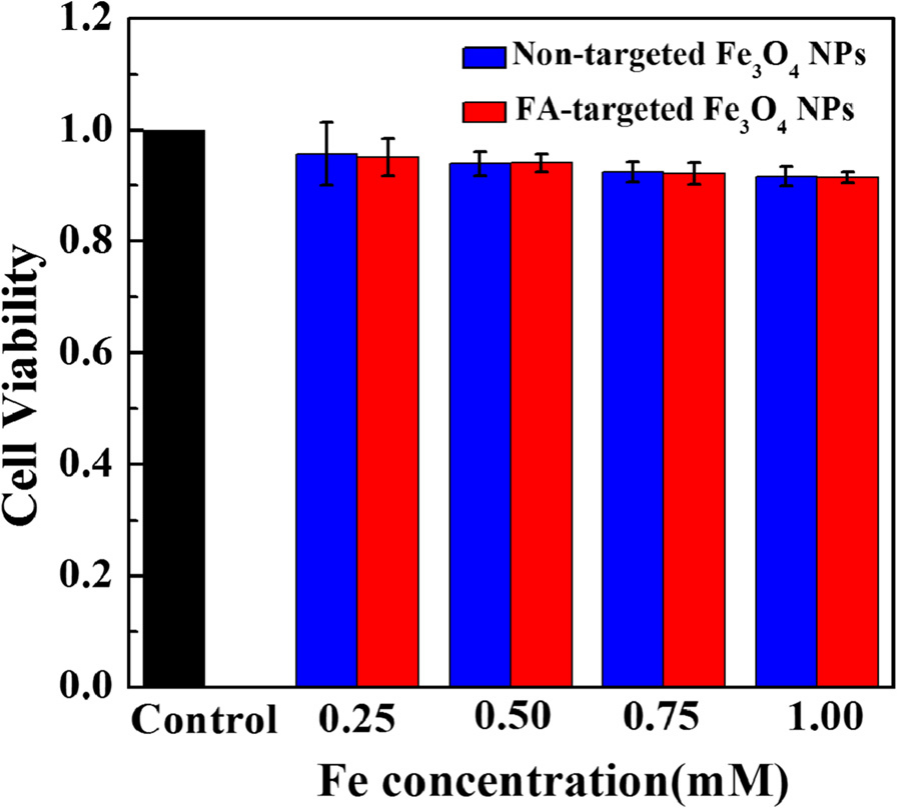
MTT assay of Skov-3 cell viability after treatment with PBS (control) and the FA-targeted Fe_3_O_4_ NPs or non-targeted Fe_3_O_4_ NPs at the Fe concentration of 0.25–1.00 mM for 24 h

It is important to assess the potential cytotoxicity of Fe_3_O_4_ NPs before their biomedical applications. After incubation of Skov-3 cells with FA-targeted Fe_3_O_4_ NPs or non-targeted Fe_3_O_4_ NPs at the Fe concentrations of 0.25, 0.50, 0.75 or 1.00 mM for 24 h, cell viability was assessed with the MTT assay (Fig. 4). The cell viability did not change significantly after treatment with either kind of Fe_3_O_4_ in the studied concentration range when compared with cell viability of control cells treated with PBS (*n* = 3). The results of MTT assay indicated the low cytotoxicity of the prepared Fe_3_O_4_ NPs, which is very important for their further in vivo applications.

### Cellular uptake of FA-targeted Fe_3_O_4_ NPs and non-targeted Fe_3_O_4_ NPs

**Fig. 5:**
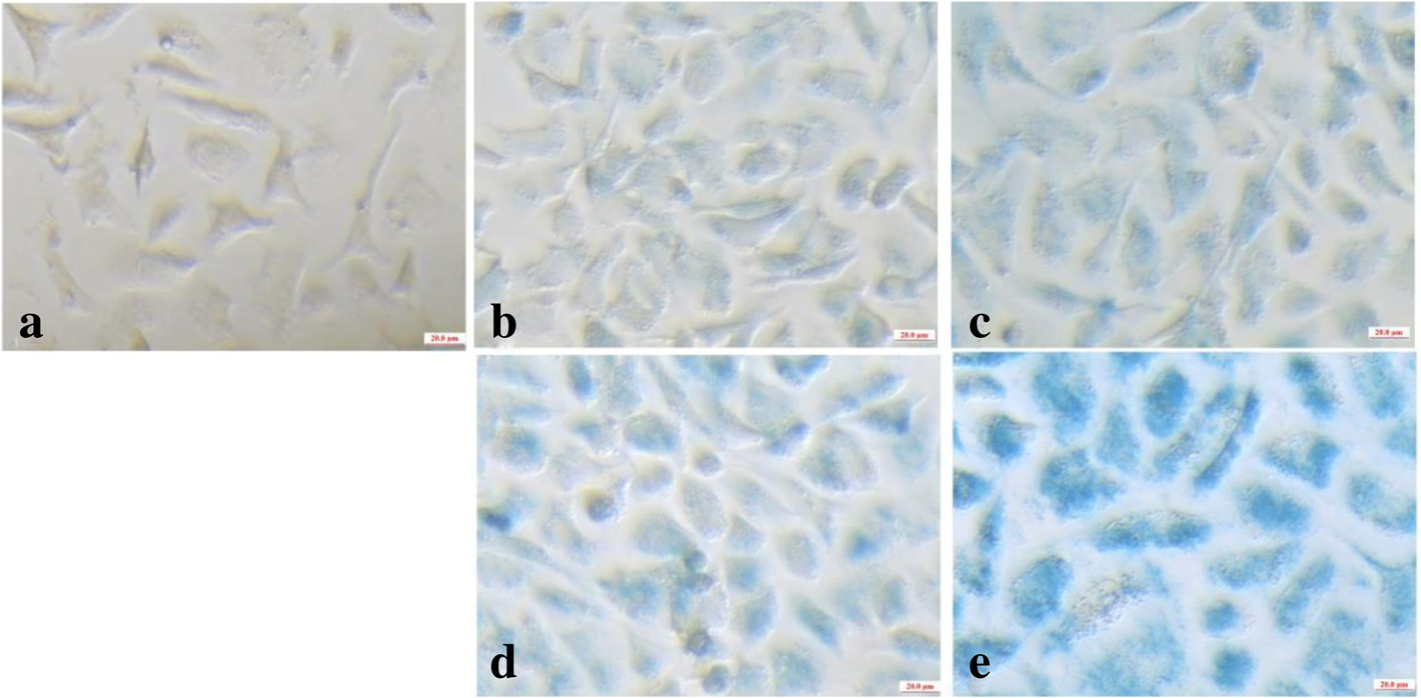
Prussian blue-stained Skov3 cells incubated with PBS (**a**), non-targeted Fe_3_O_4_ NPs (**b**, **c**) and FA-targeted Fe_3_O_4_ NPs (**d**, **e**) in given Fe concentration of 0.2 mM (**b**, **d**) and 0.4 mM (**c**, **f**). Scale bar = 20 μm

Prussian blue staining was carried out to assess the cellular uptake of FA-targeted Fe_3_O_4_ NPs and non-targeted Fe_3_O_4_ NPs by Skov-3 cells. The results showed that the uptake of iron component correlated directly with Fe concentration, and the cells appeared dark blue compared with the control cells (Fig. 5). The Prussian blue staining also demonstrated that Skov-3 cells treated with FA-targeted Fe_3_O_4_ NPs had more obvious blue staining than the cells treated with non-targeted Fe_3_O_4_ NPs at the same Fe concentration. We interpreted these results as evidence that the FA-targeted Fe_3_O_4_ NPs had a higher affinity to Skov-3 cells than the non-targeted Fe_3_O_4_ NPs.

**Fig. 6:**
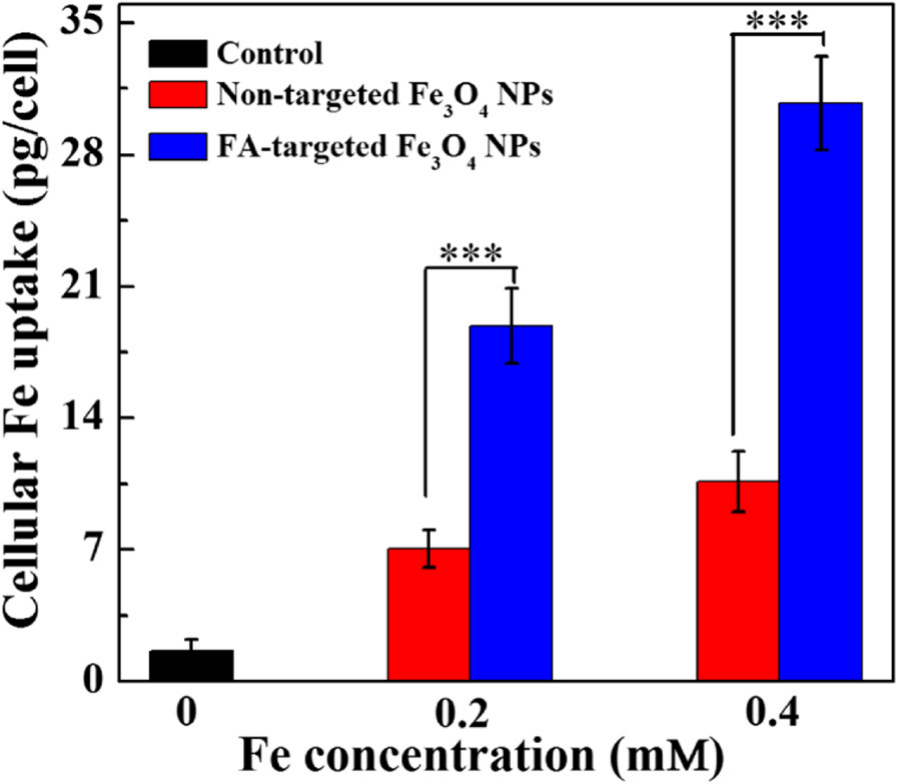
Cellular uptake assay of the Skov3 cells after treatment with FA-targeted Fe_3_O_4_ NPs or non-targeted Fe_3_O_4_ NPs at Fe concentration of 0.2 and 0.4 mM

To further document that FA could facilitate the specific uptake of FA-targeted Fe_3_O_4_ NPs by Skov-3 cells, the cells were incubated with FA-targeted Fe_3_O_4_ NPs or non-targeted Fe_3_O_4_ NPs at the Fe concentrations of 0.2 and 0.4 mM for 4 h. Then the Fe concentration in the cells was analyzed by ICP-OES. As shown in Fig. 6, the cellular uptake increased as a function of Fe concentration for both Fe_3_O_4_ NPs. At the same Fe concentration, the Skov-3 cells treated with FA-targeted Fe_3_O_4_ NPs displayed much higher uptake than those treated with non-targeted Fe_3_O_4_ NPs (*n* = 3). These results indicated that the FA-targeted Fe_3_O_4_ NPs can be specifically taken up by the Skov-3 cells overexpressing FA receptors via ligand-mediated endocytosis pathway.

### In vivo targeted MR imaging

**Fig. 7:**
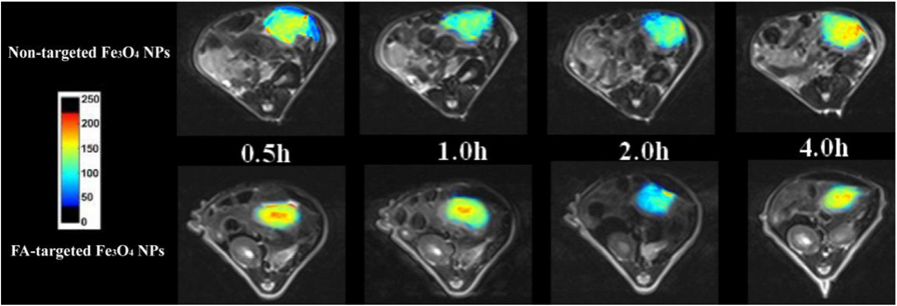
In vivo MR imaging of intraperitoneal tumor after intravenous injection of FA-targeted Fe_3_O_4_ NPs or non-targeted Fe_3_O_4_ NPs (0.6 mg Fe) at different time points

**Fig. 8:**
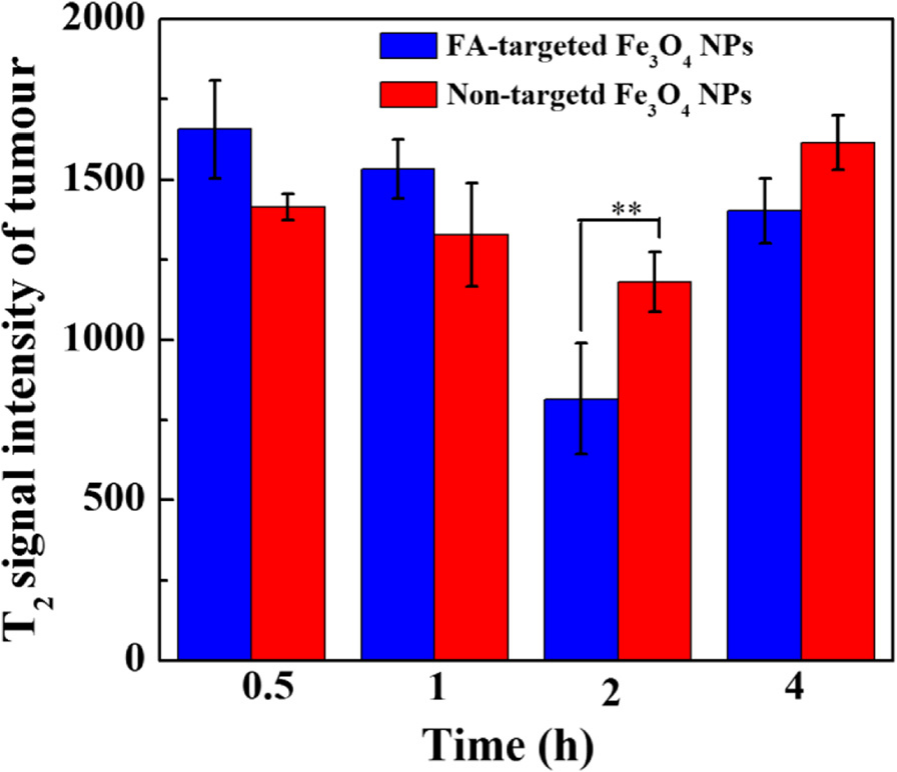
Measurements of T_2_ signals intensity of intraperitoneal tumor in nude mice after intravenous injection of FA-targeted Fe_3_O_4_ NPs or non-targeted Fe_3_O_4_ NPs (0.6 mg Fe) at different time points

After intravenous injection of the FA-targeted Fe_3_O_4_ NPs or non-targeted Fe_3_O_4_ NPs into the mice bearing intraperitoneal ovarian tumors, MR scanning was performed. The tumor MR signal for the mice injected with both particles gradually decreased with time after injection (Fig. 7). In the MR images, we can see the contrast enhancement was highest at 2 h post injection. After that, the tumor signal recovered because of further metabolism. Quantitative analysis of the T_2_ signal intensity of solid tumors at various time points revealed that the lowest signal intensity occurred at 2 h after injection with both the FA-targeted Fe_3_O_4_ NPs and non-target Fe_3_O_4_ NPs (Fig. 8). The T_2_-weighted signal intensity of the lesions at 0.5, 1, 2, and 4 h post injection was 1666 ± 152, 1534 ± 92, 749 ± 56 and 1402 ± 102 for the FA-targeted Fe_3_O_4_ NPs group and 1414 ± 42, 1328 ± 162, 1181 ± 93 and 1615 ± 84 for the non-targeted Fe_3_O_4_ NPs group, respectively (Fig. 8). It should be noted that the T_2_ signals intensity of the mice treated with FA-targeted Fe_3_O_4_ NPs was significantly lower than that of the mice treated with non-targeted Fe_3_O_4_ NPs at 2 h post injection (*P* = 0.002, *n* = 3). This results suggested that the prepared FA-targeted Fe_3_O_4_ NPs have a great potential to be used as contrast agents for targeted MR imaging to diagnosis the ovarian tumors.

## Discussion

In this study, we report our preliminary experience in imaging human ovarian cancer in the xenograft tumor model by using FA-targeted Fe_3_O_4_ NPs as contrast agents. Owing to the good contrast enhancement and low cytotoxicity, the FA-targeted Fe_3_O_4_ NPs can detect the ovarian cancer tissues planted in the abdominal cavity of nude mice at in vivo levels. Our results indicated that FA-targeted Fe_3_O_4_ NPs hold promise for being effective magnetic molecular probes for detecting tumor tissues in gynecologic cancer.

Ovarian cancer is the most malignant gynecological tumor and therefore deserves extensive basic and clinical research in the quest for early diagnostic tests and effective treatments [24–27]. Fe_3_O_4_ NPs are low-toxic and eventually biodegrade to form blood hemoglobin [14], and they have been used for liver imaging since the 1900s [28]. With recent advances in nanotechnology and nanoscience [29–32], various polymers have been coated onto the surface of Fe_3_O_4_ NPs to improve their stability and decrease their uptake by the reticuloendothelial system [16, 33, 34]. Numerous studies on application of NPs in biomedical imaging have been reported in recent decades [35–40], but few have examined application of the particles in ovarian cancer.

In our previous work, we demonstrated that FA-targeted Fe_3_O_4_ NPs have good water-dispersibility, colloidal stability and fairly high relaxivity [23]. In addition, the particles have excellent hemocompatibility and cytocompatibility in the studied range of concentrations. We found that FA-targeted Fe_3_O_4_ NPs had excellent binding specificity to a human cervical cancer cell line (HeLa cells) overexpressing FA receptors via an active FA targeting pathway. In the present study, we found excellent lesion targeting ability of the FA-targeted Fe_3_O_4_ NPs to ovarian cancer in the T_2_-weighted MR imaging, which may be attributed to the following aspects: First, although the mean size of the FA-targeted Fe_3_O_4_ NPs was small (9.2 ± 1.7 nm), the particles had a very high r_2_ relaxivity coefficients (475.92 mM^−1^s^−1^), which is much higher than those of other reported Fe_3_O_4_ NPs [33, 40]. This feature made the particles more sensitive to magnetic susceptibility effects. Second, the presence of FA on the surface of the Fe_3_O_4_ NPs increased their ability to target tumor tissues. Third, the passive enhanced permeability and retention effect into solid tumors may also facilitate the specific MR imaging of tumors [36].

Human ovarian cancers are located deep in the pelvic space [41]. An ideal humanized xenograft mouse model of ovarian cancer would simulate the true microenvironment for tumor angiogenesis [24, 42–44]. Thus, in the present study, we implanted the tumor cells in the abdomen rather than in subcutaneous sites, believing that the intraperitoneal location would reflect the hemodynamic condition of ovarian cancer in humans-at least more accurately than would a subcutaneous site, as has been often used [10–12, 25]. Our results corroborated this point: both targeted and non-targeted particles were evident by T_2_-weithted MR imaging at 2 h after injection in abdominal tumors compared with 1 h in subcutaneous tumors [23], perhaps because more time was needed for Fe_3_O_4_ NPs to reach the deep abdominal tumors in sufficient concentration to be evident on T_2_-enhanced imaging. We must confess that the T_2_ signal intensity in MR images also achieve the lowest point at 2 h post injection of non-targeted Fe_3_O_4_ NPs, which may be due to the enhanced permeability and retention (EPR) effect (passive uptake) as well documented in solid tumors [14, 36, 37]. However, both in vitro and in vivo imaging results (as shown in Figs. 5 and. 7) proved FA-targeted ligands can enable the tumor uptake through more active pathway, thus making the tumors look like more dark compared with non-FA targeted group.

Further, we also found that, after injection of Fe_3_O_4_ NPs in the nude mice, the tumor T_2_ signal intensity had reverted to pre-injection intensity after 4 h, a little earlier than that we had found previously in subcutaneous tumors [23]. We also acknowledge that the injection of suspensions of tumor cells into the mice is different from the formation of tumors in the natural environment.

Other methods for imaging detection of ovarian cancers have been described. Hensley, et al [45] described a dual MR-fluorescence molecular tomography approach, with commercially available fluorescent molecular imaging probes for the detection and quantification of tumor-associated metabolites in ovarian carcinomas in a transgenic mouse model of epithelial ovarian cancer. The authors concluded that the combination of in vivo molecular and MR imaging can effectively detect orthotopic ovarian tumors and their response to therapy [25]. In another study, Satpathy, et al [24] reported that in an orthotopic human ovarian tumor xenograft model, HER-2- targeted magnetic NPs labeled with a near infrared dye (NIR-830) were specifically delivered into primary and disseminated ovarian tumors, enabling optical and MR imaging of tumors as small as 1 mm in the peritoneal cavity. The authors designed the non-conjugated magnetic NPs with 14 ± 3.4 nm diameter and targeted-conjugated magnetic NPs with 22.9 ± 4.8 nm diameter, respectively [24]. However, they did not report the exact MR acquisition time point, which we believe is crucial for tumor imaging, especially for magnetic NPs.

Our study also has some limitations. First, by 2 weeks after injection of ovarian cancer cells into the peritoneal cavity, the tumors often had become large (average diameter about 5 mm) with isointensity signals on T_2_-weighted MR imaging making them easily detectable and distinct from surrounding tissues, which had mostly hyperintensity signals. However, tumors at an earlier stage or smaller might not be detected because of overlapping neighboring organs (such as gut, kidney, or bladder) and background tissues. Perhaps the specificity and sensitivity could be improved by the use of bimodal magnetic nanoprobes with fluorescent materials incorporated into Fe_3_O_4_ NPs. Second, since FA receptors are overexpressed in most malignant tumors, the FA targeting ligand we used may not be specific for detecting ovarian cancer. Further studies should be conducted to image ovarian cancer with targeting motifs that may be more specific.

## Conclusion

In summary, this study demonstrated that the prepared FA-targeted Fe_3_O_4_ NPs can bound specifically in vitro to the FA receptors overexpressed human serous ovarian cells without inducing cytotoxicity. Used as T_2_-negative contrast agents in MR imaging, the particles also localized to intraperitoneal human ovarian cancer tissues in a xenograft tumor model. Importantly, the tumor can be detected more obviously after the mice were injected with FA-targeted Fe_3_O_4_ NPs than non-targeted Fe_3_O_4_ NPs. Thus, FA-targeted Fe_3_O_4_ NPs hold promise that they may be multifunctional nanoprobes for the diagnosis and treatment of ovarian cancer.

### Ethics approval

All the animal experiments were performed according to protocols approved by the Ethical Committee of Obstetrics and Gynecology Hospital ([2007]-No. 6), Fudan University.

### Consent for publication

Not applicable.

## Abbreviations

DMSO: dimethyl sulfoxide
EPR: enhanced permeability and retention
FA: folic acid
FBS: fetal bovine serum.
Fe_3_O_4_: iron oxide
ICP-OES: Leeman prodigy inductively coupled plasma-optical emission spectroscopy
MR: magnetic resonance
MTT: 3-(4,5-dimethylthiazol-2-yl)-2,5-diphenyltetrazolium bromide
NPs: nanoparticles
PBS: phosphate-buffered saline
PEG: polyethylene glycol
PEI: polyethyleneimine
TEM: transmission electron microscopy
